# Persistence with antihypertensives in uncomplicated treatment-naïve very elderly patients: a nationwide population-based study

**DOI:** 10.1186/s12872-017-0665-4

**Published:** 2017-08-24

**Authors:** Kyung Hee Choi, Yun Mi Yu, Young-Mi Ah, Min Jung Chang, Ju-Yeun Lee

**Affiliations:** 1 College of Pharmacy, Sunchon National University, 255 Jungang-ro, Suncheon, Jeollanam-do 57922 South Korea; 2 College of Pharmacy & Research Institute of Pharmaceutical Sciences, Seoul National University, 103 Daehak-ro, Jongno-gu, Seoul, 03080 South Korea; 3 College of Pharmacy, Institute of Pharmaceutical Science and Technology, Hanyang University, 55 Hanyangdaehak-ro, Sangnok-gu, Ansan, Gyeonggi-do 15588 South Korea; 4 College of Pharmacy and Yonsei Institute of Pharmaceutical Sciences, Yonsei University, Incheon, 21983 South Korea; 5 College of Pharmacy, Chonnam National University, Gwang-Ju, 61186 South Korea

**Keywords:** Hypertension, Treatment persistence, Adherence, Aged, Very elderly

## Abstract

**Background:**

Limited studies have evaluated the medication-taking behavior in very elderly hypertensive patients. The aim of this study was to evaluate the persistence and adherence with antihypertensive agents in treatment-naïve patients, along with other related factors, according to age.

**Methods:**

Adult (19–64 years), elderly (65–79 years), and very elderly (≥80 years) uncomplicated hypertensive patients starting antihypertensive monotherapy were identified from the National Health Insurance claims database. The first-year treatment persistence and adherence rates measured using the medication possession ratio were assessed and compared in these three age cohorts.

**Results:**

After propensity score matching, three age cohorts with 6689 patients each were assembled from 228,925 uncomplicated hypertensive patients who began antihypertensive monotherapy in 2012. The treatment persistence and adherence rates over the first year were the lowest in the very elderly (59.5% and 62.8%, respectively) and highest in the elderly (65.2% and 67.9%, respectively) patients among the three age cohorts (*p* < 0.001). The adjusted risk for treatment non-persistence was significantly higher in the very elderly (adjusted hazard ratio, 1.20; 95% confidence interval, 1.13–1.27) compared with the elderly. Having more comorbidities, being a beneficiary of medical aid, and having a diagnosis of dementia were unique positive predictors for treatment persistence in the very elderly, along with common predictors such as female sex, dyslipidemia, and an initially chosen antihypertensive therapeutic class other than beta blockers and thiazide diuretics.

**Conclusions:**

Very elderly patients were less likely to continue antihypertensive therapy over the first year compared with their younger counterparts. Our findings suggest that a low comorbidity index and lack of medical aid support negatively affect the treatment persistence in this population.

## Background

Hypertension is a major health problem worldwide and requires long-term management and continuous therapy. The prevalence among aged people is substantial; approximately 80% of very elderly patients (defined as those aged over 80 years) have been reported to have hypertension [1]. Similarly, according to a report from the National Health Service in England, the prevalence of hypertension was 66.6% in the population aged over 75 years, as compared with 29.6% in the overall adult populations in 2014 [2]. As the elderly population increases (especially the very elderly population) [3], the number of patients with chronic diseases requiring long-term care is also increasing. As a result, the healthcare expenditures are increasing worldwide, representing an important social and public health issue [4].

Effective management of hypertension in the elderly is vital for preventing disease progression and related comorbidities and for reducing health care costs [5, 6]. Although hypertension is a principal cause of cardiovascular and cerebrovascular events and is associated with increased mortality [6, 7], before the publication of the Hypertension in the Very Elderly Trial, the benefits of treatment in very elderly patients with hypertension were controversial. This trial confirmed that treatment of hypertension reduced mortality even in the very elderly, indicating its importance [8, 9].

Age has been considered a potential factor that can influence treatment persistence and adherence [10, 11], and previous studies have mainly focused on elderly populations [12, 13]. In 2008, van Wijk et al. measured persistence with antihypertensive treatment in elderly populations in the US, Canada, and the Netherlands, and found similar patterns of persistence among the three countries [12].

The patterns of medication persistence in Asian population may differ from those in Western countries because of differences in race, healthcare systems, and socioeconomic environment [14, 15]. Different racial and ethnic groups show diverse perceptions and attitudes towards health and medicines, which may affect adherence to therapy [16]. Various reimbursement coverages, drug prices, and financial barriers depending on country-specific healthcare system and socioeconomic environment have been reported to affect medication persistence [14, 15, 17].

However, very few studies have evaluated hypertensive treatment persistence and adherence in very elderly vs. elderly hypertensive patients in Asia, including Korea. In Korea, the National Health Insurance, a public insurance program operated by the Ministry of Health and Welfare, covers most of the Korean population claims data, including patient data such as the diagnosis, prescription medication, and medical costs, collected by each medical institution. Using these nationwide healthcare claims data in Korea, this study aimed to compare the antihypertensive treatment persistence and adherence patterns in very elderly patients with uncomplicated hypertension to those of matched elderly and adult patients. In addition, we also aimed to identify the factors associated with non-persistence in the very elderly population.

## Methods

### Data source and cohort definition

For this retrospective cohort study, we used the Korean National Health Insurance claims database from January 2011 to December 2013, provided by the Health Insurance Review Agency following data anonymization.

We identified newly diagnosed uncomplicated hypertensive adult patients who started antihypertensive monotherapy in 2012. The index date was defined as the date of the first antihypertensive prescription in 2012. Patients with a diagnosis code of hypertension (International Classification of Diseases [ICD-10] codes I10–13, I15) or who had been prescribed any antihypertensive medication within 1 year before the index date were excluded to ensure the inclusion of only treatment-naïve hypertensive patients. Patients who had been previously diagnosed with the following comorbid conditions were also excluded: cardiovascular disease (I20-I25, I30-I52, Z95), cerebrovascular disease (G45, I60-I69), peripheral vascular disease (I7X), renal disease (N03-N05, N18, N19, Z49, Z94.0, Z99.2), diabetes mellitus (E08-E11, E13), and pregnancy (O00-O9A) [18]. In addition, to evaluate the patients’ medication-taking behavior, we excluded patients who had been prescribed only one dose of antihypertensive medication or who had taken the medications for a period of less than 7 days, as well as those who had been hospitalized for more than 7 days within one year from the index date. The patients were followed for one year from the index date and were excluded if their claims data were discontinued before the end of the follow-up period.

The patients were classified into three age categories: adult (19–64 years), elderly (65–79 years), and very elderly (≥80 years). The elderly and adult cohorts were constructed using propensity matching with common reference cohorts of the very elderly [18]. The matching variables included sex, the health insurance type, and the Charlson Comorbidity Index [19]. The Charlson Comorbidity Index score, a prognostic comorbidity rating score calculated based on 19 disease states each assigned with a score of 1, 2, 3, or 6, corresponding to the risk of mortality, was assessed using the ICD-10 codes to evaluate the patient’s disease burden for 1 year before the index date [19]. The prescribed antihypertensive medications used as the initial therapy were classified by their therapeutic class: angiotensin receptor blockers (ARBs), angiotensin converting enzyme inhibitors (ACEIs), selective beta blockers (BBs), dihydropyridine calcium channel blockers (CCBs), thiazide type diuretics (Ds), alpha blockers (ABs), and others.

The study protocol was approved by the Institutional Review Board (IRB) of Seoul National University (IRB number: P01–201406-SB-03-02). A full ethical review was made for all procedures following the protocol approved by the IRB. The board waived the need for informed consent because only de-identified information was used, without linkable data elements.

### Treatment persistence and adherence

Treatment persistence was measured by the treatment duration, defined as the number of days from the index date to the discontinuation of any antihypertensive treatment or the first appearance of a 60-day prescription gap.

To assess treatment adherence, we used the medication possession ratio (MPR), calculated as the sum of days covered by any antihypertensive medication one year from the index date divided by 365 days. An MPR ≥0.8 was considered adherent [20].

To examine the patients’ age as a factor affecting treatment non-persistence, we calculated the adjusted hazards for treatment non-persistence for the very elderly and elderly cohorts compared with the reference group of adults. We also calculated the adjusted hazard for the very elderly with elderly patients as the reference.

### Factors associated with treatment non-persistence

We assessed the patient sex, Charlson Comorbidity Index score, insurance type, specific underlying diseases (dyslipidemia, dementia, and depression), and the initial therapeutic class of the index therapy as factors potentially affecting treatment non-persistence in the three cohorts. The Charlson Comorbidity Index scores were obtained using the diagnosis codes.

### Statistical analysis

Demographic and clinical characteristics of the three cohorts are presented with descriptive statistics, such as the mean, standard deviation (SD), and percentage. Comparisons of the continuous and discrete data among the three cohorts were performed using analysis of variance (ANOVA) and chi-squared tests, respectively. We considered a *p* value ≤0.05 to be statistically significant and adjusted this value using Bonferroni correction.

The treatment persistence rates over the first year stratified by the three cohorts were compared using the log-rank test and are presented as adjusted Kaplan-Meier curves. Multivariate Cox-proportional hazards models were used to evaluate the influence of the potential risk factors on treatment non-persistence. The degree of association is described by the adjusted hazard ratio (aHR) and corresponding 95% confidence interval (CI). Data management and statistical analyses were performed using SAS 9.3 (SAS Institute, Inc., Cary, NC).

## Results

### Patient selection

We identified 228,925 uncomplicated, treatment-naïve hypertensive patients who began antihypertensive monotherapy in 2012. After excluding patients who were followed-up for less than one year (*n* = 9364), a total of 219,561 patients remained. Of these, 6803 patients (3.1%) were classified as very elderly and 37,982 patients (17.3%) as elderly. After propensity score matching for sex, Charlson Comorbidity Index score, and health insurance type, 6689 patients were included in each of the three cohorts.

### Demographic and clinical characteristics

The mean (± SD) ages of the adult, elderly, and very elderly cohorts were 50.4 (± 8.9), 70.9 (± 4.1), and 84.3 (± 4.0) years, respectively. Females comprised 72.6% of the population in all three cohorts. The mean (± SD) Charlson Comorbidity Index score at baseline was 0.5 (± 0.9), and 7.2% of all patients were receiving medical aid. The number of concomitant medications was the highest in the very elderly cohort and lowest in the adult cohort (*p* < 0.05). Compared with the other two cohorts, the very elderly cohort had the highest proportion of dementia patients and lowest proportion of patients with dyslipidemia (*p* < 0.0001). Depression was more prevalent in the adult cohort than in the other cohorts (*p* < 0.0001). Overall, the most frequent therapeutic class of index therapy was CCBs (48.9%), followed by ARBs (32.0%), BBs (10.0%), and Ds (6.0%). While this trend was also observed in the very elderly and elderly cohorts individually, ARBs were somewhat more prevalent than CCBs in the adult cohort (Table 1).

**Table 1:** Demographic and clinical characteristics of the propensity-matched cohort

Characteristics	Total (*n* = 20,067)	Adults ^a^ (*n* = 6689)	Elderly ^b^ (*n* = 6689)	Very elderly ^c^ (*n* = 6689)	*p* value^*^	Post hoc^†^
Age, years, mean (SD)	68.5 (15.2)	50.4 (9.0)	70.9 (4.1)	84.3 (4.0)	<0.0001	a < b < c
Sex, male, n (%)	5502 (27.4)	1834 (27.4)	1834 (27.4)	1834 (27.4)	1.000	
Charlson comorbidity index score, mean (SD)	0.5 (0.9)	0.5 (0.9)	0.5 (0.8)	0.5 (0.9)	0.948	
0	12,780 (63.7)	4258 (63.7)	4264 (63.8)	4258 (63.7)	1.000	
≥ 1	7287 (36.3)	2431 (36.3)	2425 (36.3)	2431 (36.3)	0.992	
Health insurance type						
Health insurance	18,621 (92.8)	6207 (92.8)	6207 (92.8)	6207 (92.8)	1	
Medical aid	1446 (7.2)	482 (7.2)	482 (7.2)	482 (7.2)		
Number of concomitant medications, mean (SD)	3.3 (2.8)	2.7 (2.4)	3.4 (2.8)	3.7 (3.0)	<0.0001	a < b < c
Underlying disease, n (%)						
Dementia	277 (2.1)	24 (0.2)	59 (0.9)	194 (2.9)	<0.0001	a < b < c
Depression	639 (4.6)	440 (3.2)	104 (1.6)	95 (1.4)	<0.0001	a > b,c
Dyslipidemia	2496 (16.3)	1963 (12.8)	366 (5.5)	167 (2.5)	<0.0001	a > b > c
Therapeutic class of index therapy, n (%)						
Dihydropyridine calcium channel blockers	9807 (48.9)	2619 (39.2)	3374 (50.4)	3814 (57.0)	<0.0001	<0.0001
Angiotensin receptor blockers	6419 (32.0)	2722 (40.7)	2077 (31.1)	1620 (24.2)		
Selective beta blockers	2009 (10.0)	900 (13.5)	620 (9.3)	489 (7.3)		
Thiazide type diuretics	1212 (6.0)	324 (4.8)	412 (6.2)	476 (7.1)		
Alpha blockers	359 (1.8)	29 (0.4)	128 (1.9)	202 (3.0)		
Angiotensin converting enzyme inhibitors	250 (1.2)	84 (1.3)	78 (1.2)	88 (1.3)		
Others	11 (0.1)	11 (0.2)	0 (0.0)	0 (0.0)		

^*^Analysis of variance test or chi-squared test of the three groups

^†^Bonferroni correction (*p* < 0.015) with student’s t-test or chi-squared test

^a^Adult cohort ^b^Elderly cohort ^c^Very elderly cohort

### Treatment persistence and adherence

**Fig. 1:**
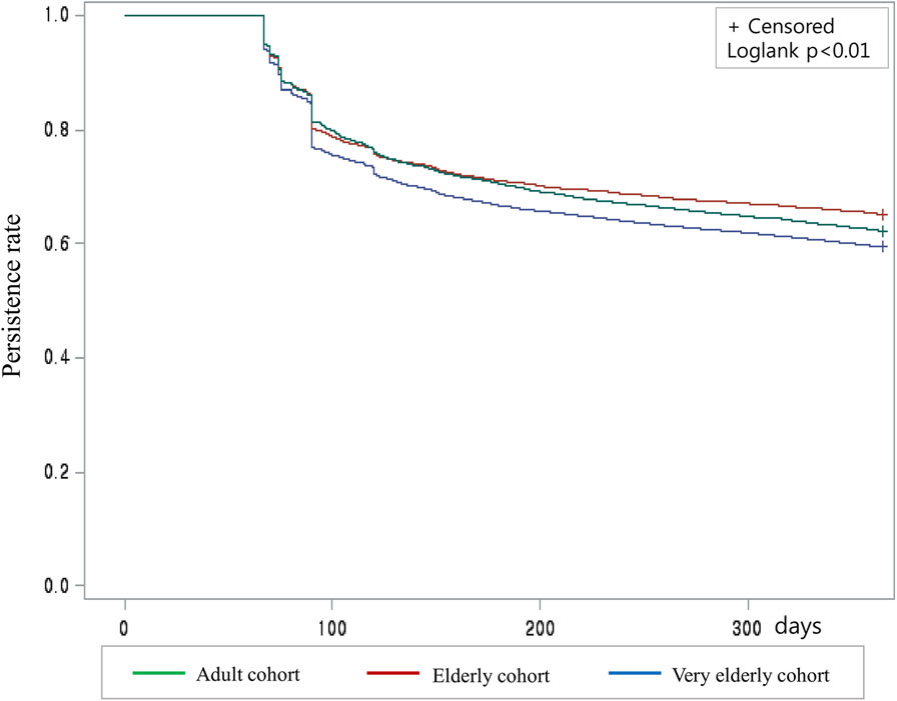
Treatment persistence rate over the first year among adult, elderly and very elderly cohorts (*n* = 20,067). Green line: adults (19–64 years); Red line: elderly (65–79 years); Blue line: very elderly (≥80 years)

The very elderly cohort showed a significantly shorter treatment duration than the other two cohorts (*p* < 0.05, Table 2). Although similar proportions of patients continued antihypertensive treatment after the first three months, the proportion of patients who remained on any antihypertensive treatment at the end of the first year was significantly lower in the very elderly cohort (59.5%) than in the elderly cohort (65.2%, *p* < 0.001). Adjusted Kaplan-Meier analysis of the treatment persistence rate over the first year also revealed lower treatment persistence in the very elderly cohort compared to in the other two cohorts (Fig. 1).

**Table 2:** Comparison of treatment persistence and adherence among propensity-matched cohorts

Variables	Adults ^a^ (*n* = 6689)	Elderly ^b^ (*n* = 6689)	Very elderly ^c^ (*n* = 6689)	*p*-value^*^	Post hoc^†^
Treatment duration, days, mean (SD)	277.1 (122.8)	280.6 (123.3)	267.6 (127.2)	<0.0001	a,b > c
Treatment persistence rate, n (%)					
at the first 3 months	5753 (86.0)	5764 (86.2)	5663 (84.7)	0.024	b > c
at the first 6 months	4731 (70.7)	4754 (71.1)	4473 (66.9)	<0.0001	a, b > c
at the first 9 months	4422 (66.1)	4532 (67.8)	4207 (62.9)	<0.0001	a, b > c
at the first 12 months	4179 (62.5)	4364 (65.2)	3979 (59.5)	<0.0001	b > a > c
Medication possession ratio, mean (SD)	0.76 (0.30)	0.79 (0.29)	0.75 (0.30)	<0.0001	b > a, c
Treatment adherence rate, n (%)	4290 (64.1)	4542 (67.9)	4135 (62.8)	<0.0001	b > a > c

^*^Analysis of variance test or chi-squared test of the three groups

^†^Bonferroni correction (*p* < 0.015) with student’s t-test or chi-squared test

^a^Adult cohort ^b^Elderly cohort ^c^Very elderly cohort

The adherence rate over one year showed a similar pattern, with the lowest rate observed in the very elderly cohort (62.8% vs. 64.1% and 67.9% in the adult and elderly cohorts, respectively, *p* < 0.001 for both) (Table 2).

After adjusting for sex, insurance type, comorbidities, underlying disease, and initial therapeutic class of index therapy, the likelihood of discontinuing antihypertensive treatment was significantly higher in the very elderly patients (aHR, 1.20; 95% CI, 1.13–1.27) compared with in the elderly patients. However, the adjusted risk of treatment non-persistence in the very elderly (aHR, 0.99; 95% CI, 0.94–1.05) was similar to that of adults (aHR, 0.99; 95% confidence interval (CI), 0.94–1.05), while elderly patients exhibited a significantly lower risk than adults (aHR, 0.84; 95% CI, 0.79–0.89).

### Predictors of treatment non-persistence

Table 3 shows the predictors of treatment non-persistence in the three cohorts. Although the presence of comorbidities was not significantly associated with treatment persistence in uncomplicated adult or elderly hypertensive patients, patients with at least one comorbidity were more likely to continue treatment in the very elderly cohort (aHR, 0.93; 95% CI, 0.87–0.98). Among the very elderly, patients who received medical aid were more likely to persist with the treatment (aHR, 0.83; 95% CI, 0.71–0.98), while in the adult cohort, those receiving medical aid were at higher risk of non-persistence (aHR, 1.21; 95% CI, 1.06–1.39). Patients with dementia showed a lower risk of discontinuing treatment only in the very elderly cohort (aHR, 0.68; 95% CI, 0.52–0.90). Finally, in terms of the initial therapeutic classes of drugs, only Ds and BBs were associated with treatment non-persistence in elderly and very elderly patients (Table 3).

**Table 3:** Predictors for antihypertensive treatment non-persistence in three propensity-matched cohorts ^a^

Characteristics	Adults (*n* = 6689)		Elderly (*n* = 6689)		Very elderly (*n* = 6689)	
	aHR	95% CI	aHR	95% CI	aHR	95% CI
Sex						
Male (reference)	1.00		1.00		1.00	
Female	0.90	0.82–0.98	0.92	0.84–1.01	0.85	0.78–0.93
Charlson comorbidity index score						
0 (reference)	1.00		1.00		1.00	
≥ 1	0.95	0.87–1.03	0.98	0.92–1.04	0.93	0.87–0.98
Health insurance type						
Health insurance (reference)	1.00		1.00		1.00	
Medical aid	1.21	1.06–1.39	1.07	0.92–1.26	0.83	0.71–0.98
Underlying disease risk factor						
Dementia	1.66	0.93–2.97	1.15	0.75–1.77	0.68	0.52–0.90
Depression	1.11	0.95–1.28	1.36	1.00–1.85	0.91	0.63–1.32
Dyslipidaemia	0.64	0.59–0.71	0.75	0.61–0.92	0.75	0.57–0.98
Therapeutic class of index therapy						
Angiotensin receptor blockers (reference)	1.00		1.00		1.00	
Dihydropyridine calcium channel blockers	1.12	1.02–1.22	1.03	0.93–1.13	1.04	0.94–1.14
Selective beta blockers	1.93	1.73–2.16	1.58	1.37–1.82	1.29	1.11–1.51
Thiazide diuretics	4.04	3.49–4.67	2.87	2.48–3.33	1.74	1.51–2.02
Alpha blockers	1.90	1.15–3.15	1.23	0.92–1.65	1.03	0.81–1.30
Angiotensin converting enzyme inhibitors	1.42	1.03–1.97	1.14	0.77–1.68	0.96	0.68–1.37
Others	2.39	1.18–4.88				

*Abbreviations*: *aHR* adjusted hazard ratio; *CI* confidence interval

^a^Predictors for antihypertensive treatment non-persistence were identified using a multivariate Cox-proportional Hazard model

## Discussion

To our knowledge, this is the first nationwide population-based study focused on antihypertensive medication persistence and adherence in a very elderly population at the time of initiation of antihypertensive monotherapy in Korea. We found that very elderly patients were more likely to discontinue antihypertensive treatment than elderly or adult patients, and that the predictors of treatment non-persistence in the very elderly differed from those in the other age groups. The one-year treatment persistence rate in very elderly patients was significantly lower than that in elderly patients (59.5% vs. 65.2%, *p* < 0.001); after adjustment for potential confounding variables, very elderly patients remained at elevated risk of treatment non-persistence compared with elderly patients (aHR, 1.20; 95% CI, 1.13–1.27). Despite the statistically significant differences in treatment persistence and adherence among the three age cohorts, the one-year treatment adherence was more than 60% in all three groups. These results corroborate the findings from other studies conducted in Western countries [12, 13]. In 2008, van Wijk et al. reported that approximately 25% of elderly patients studied were non-persistent, defined as a treatment gap of at least 180 days during the first year of treatment, with little difference among the three examined countries (the US, Canada, and the Netherlands) [12]. In 2014, Tu et al. found that 64.6% of elderly patients in Canada were therapy-persistent for over one year [13].

The present study found that elderly patients aged 65–79 years were more persistent with antihypertensive treatment than adults, while the very elderly showed poor treatment persistence. This result is consistent with previously published results from Canada [13] and Sweden [21], which showed reduced odds of medication persistence in patients older than 75 years compared to those aged 66–70 years. However, other previous studies that have investigated the impact of aging on treatment persistence have reached conflicting results [22, 23]. These discrepancies may be partially explained by differences in age composition, reference groups, and methodology used in measuring persistence, such as a prescription gap, between the studies.

Unlike in previous studies, the present study included only uncomplicated hypertensive patients. Therefore, the Charlson Comorbidity Index score was low, and two-thirds of the study patients had no comorbid conditions. The findings from previous studies investigating comorbidities as a risk factor for treatment non-persistence have been inconsistent [13, 23]. In the present investigation, we found that patients with one or more comorbidities were significantly more likely to remain treatment persistent than those without a comorbidity in the very elderly population, whereas the presence of comorbidities did not predict poor persistence in adults or in the elderly. These findings may be explained by the fact that very elderly patients with comorbidities might be more likely to continue hypertensive treatment because they require regular visits to healthcare facilities for diseases other than hypertension. Furthermore, in the very elderly population, patients with comorbidities might be more likely to continue hypertensive treatment because they require regular visits to healthcare facilities for diseases other than hypertension.

Beneficiaries of medical aid receive prescription drugs free of charge in Korea. Accordingly, in our study, being a beneficiary of medical aid was positively associated with treatment persistence in the very elderly cohort (aHR, 0.83; 95% CI, 0.71–0.98). This finding is consistent with those of several studies that reported sufficient health insurance or prescription drug coverage as the principal contributing factor to treatment persistence [10, 14, 24]. On the other hand, in our study, the insurance type was negatively associated with treatment persistence in the adult cohort (aHR: 1.21; 95% CI: 1.06–1.39). This finding may be explained by the difference in eligibility for medical aid between the adult and very elderly populations in Korea. Adults who receive medical aid in Korea tend to have a very low economic status, which is a recognized risk factor for lower treatment persistence [14, 25].

Moreover, very elderly patients diagnosed with dementia or dyslipidemia were significantly more likely to remain persistent with antihypertensive treatment. The finding of the association between dyslipidemia and treatment persistence corroborates a number of published studies [12, 23, 25, 26]. However, our finding of higher persistence in very elderly patients with dementia contrasts with the results from several previous studies reporting a higher discontinuation rate for antihypertensive treatment among patients with dementia or cognitive impairment [27, 28]. These contradictory findings are somewhat puzzling, and may be partially explained by the lower medication complexity in older adults with cognitive impairment, as observed in one previous study [29]. The care management by caregivers may also increase the treatment persistence in the very elderly with dementia [30], which is supported by the findings of recent studies that dementia was associated with the likelihoods of continuing statin therapy (aHR, 0.84; 95% CI, 0.73–0.98) and antiplatelet therapy in elderly patients after ischemic stroke [31, 32].

Ds and BBs as initial antihypertensives were negatively associated with treatment persistence, as compared with ARBs, in the very elderly cohort, whereas the rate of non-persistence in the adult cohort was slightly but significantly higher for CCB, AB, and ACEI initiators, as well as BB and D initiators, as compared with ARB initiators. The Eighth Joint National Committee guidelines do not recommend BBs for initial hypertensive therapy due to the relatively high risks of cardiovascular death, myocardial infarction, and stroke with these agents. In addition, the Canadian Institute for Health Information reported that Ds associate with the lowest persistence when newly prescribed in patients over 65 years of age [33, 34]. Marcum et al. analyzed the rate of hospitalization from adverse drug reactions in older veterans and found that BBs and Ds were associated with significantly elevated rates [35]. Thus, the low persistence commonly observed in elderly and very elderly patients when beginning a BB or D may stem from the higher risk of adverse drug reactions from these antihypertensive classes.

The increased risk of adverse drug events and inconsistency in the efficacy associated with changes in pharmacokinetic and pharmacodynamic profiles with aging also may negatively affect drug persistence particularly in the very elderly compared with the elderly patients [36, 37].

Some limitations should be considered when interpreting our findings. First, due to the nature of retrospective claims data, we could not confirm whether the patients actually took the medications that were prescribed. Therefore, our study likely overestimated the actual treatment adherence and persistence. Second, the study only included patients who started monotherapy for uncomplicated hypertension. We excluded patients who began combination therapy in order to minimize confounding by disease severity, which could not be reliably measured in our cohort. Therefore, our results may not be generalizable to patients administered combination therapy. Finally, while we measured the health insurance type as a marker of socioeconomic position, we could not consider other important socioeconomic variables due to limitations of the claims data.

## Conclusions

This study showed that very elderly patients were more likely to discontinue antihypertensive treatment when compared with elderly patients; approximately 40% of very elderly uncomplicated hypertensive patients were treatment non-persistent during the first year. In addition, this study suggests that a low comorbidity index and lack of medical aid support negatively affect the treatment persistence uniquely in this population.

## Abbreviations

Abs: Alpha blockers
ACEIs: Angiotensin converting enzyme inhibitors
aHR: Adjusted hazard ratio
ANOVA: Analysis of variance
ARB: Angiotensin receptor blockers
BBs: Selective beta blockers
CCBs: Dihydropyridine calcium channel blockers
CI: Confidence interval
Ds: Thiazide type diuretics
ICD: International classification of diseases
IRB: Institutional review board
MPR: Medication possession ratio
SD: Standard deviation
